# Germline genome editing: Moratorium, hard law, or an informed adaptive consensus?

**DOI:** 10.1371/journal.pgen.1009742

**Published:** 2021-09-09

**Authors:** Terry Kaan, Vicki Xafis, G. Owen Schaefer, Yujia Zhu, Markus K. Labude, Ruth Chadwick

**Affiliations:** 1 The University of Hong Kong, Hong Kong, China; 2 National University of Singapore, Singapore, Singapore; 3 Cardiff University, Cardiff, United Kingdom; Stanford University, UNITED STATES

## Abstract

With the development of practical means of human germline genome editing (HGGE) in recent years, there have been calls for stricter regulation and oversight over HGGE interventions with potential for heritable changes in the germline. An international moratorium has been advocated. We examine the practicality of such a proposal, as well as of a regulation through the “traditional” mechanisms of international and municipal laws. We argue that these mechanisms are unlikely to achieve their intended objectives and that the better approach is to engage the international community of stakeholders, researchers, scientists, clinicians, and other workers directly involved in the field in working toward the development of an “informed adaptive consensus”. We offer suggestions as to how this may be achieved and how existing indirect levers of regulation may be harnessed toward this end.

## Introduction

Since He Jiankui’s revelations in 2018, there have been calls for an international moratorium on human germline genome editing (HGGE) [1]. The relative technical ease with which clinical applications of HGGE can now be performed, which, for the purposes of this section, includes clinical research such as first in human trials, brings to the fore difficult ethical, legal, and social questions about how it should be appropriately regulated. In many countries, this is already directly or indirectly regulated, generally, through the licensing regime for artificial reproductive technologies [2]. Here, we aim to show first why some approaches to the regulation of HGGE would be ineffective and then identify what may be required for the successful establishment of a regulatory regime or professional guidance for HGGE. There is a pressing need to provide an understanding of the legal and regulatory environment in plain language so that scientists are well informed, given that they are likely participants in broader developments in this field, and there is an even greater pressing need to dispassionately consider solutions that will promote legally, ethically, and socially prudent actions.

We argue that none of these mechanisms alone will achieve their proposed objectives: They themselves are fraught with difficulties relating to enforcement, harmonization, and consistency of regulatory objectives between countries, especially given the need to maintain the appropriate but difficult balance between encouraging and guiding ethical scientific endeavors for the benefit of humankind, while at the same time discouraging or restricting research or clinical applications for which there is a general international consensus that such research or clinical applications are unethical or pose unacceptable and currently unknowable dangers for humanity as a whole. Scientists (and other stakeholders) have a key role to play in the development of these technologies. Instead of focusing on building high walls to contain bad actors, the collateral damage of which is to raise the administrative bar for ethical and beneficial research, and to discourage such research, scientists need to think in terms of how we can devise responsive structures that will support, guide, and encourage ethical and beneficial endeavors in this area while discouraging the dubious. Just as it is their professional and ethical responsibility to speak out if they believe that a particular line of research is not in the public interest, or is unethical (or both), we assert that if scientists believe that particular lines of HGGE can be ethically carried out with potential promise to humankind, they should likewise state their conscience. In other words, these scientists need to be advocates for the potential benefit to humanity of their work and to focus the lay mind and media on the positive and not the negative.

During the publication of this work, the World Health Organization (WHO) Expert Advisory Committee on Developing Global Standards for Governance and Oversight of Human Genome Editing released its governance framework [3] and accompanying recommendations [4]. The proposal we make in this work is in alignment with these recommendations, but, in this work, we are more specifically focused on the governance challenges of germline editing. We hope that these cumulative efforts will pave the way for greater national and international understanding and collaboration.

### Toward an informed adaptive consensus

Ethical and scientific views evolve with technology over time [5]: Given the early and rapid state of the development of the technology, a mature body of ethical and scientific consensus is currently a moving target, for which legal regulation through municipal law (i.e., national and other domestic laws such as state or regional laws) is neither appropriate nor practical. There is also the serious danger that early regulation implemented in the form of hard municipal law will have the effect of ossifying a developing consensus at an intermediate stage of development and stunt ethical research and potentially valuable scientific advances.

We suggest that the best way forward is to rally the international scientific community toward the formulation of a scientific and ethical consensus on what may be acceptable (or otherwise) at any given time in the development of the technology. We envisage that the stakeholders to such a consensus recognize that it is a consensus that is not a fixed one, but one that develops and changes over time in response to new advances and knowledge, i.e., an “informed adaptive consensus”. The development of such a flexible and responsive scientific and ethical consensus (and just as importantly, the debate that informs it) will also serve the invaluable office of providing assurance and guidance to scientists who are concerned about the ethics of their research in this area. To this end, we explore and suggest avenues for harnessing currently available levers of indirect regulation that can be used to give real teeth to such an international consensus. Finally, we suggest that with this approach, a truly mature and stable ethical and scientific consensus will come about, which can serve the purpose of guiding governments toward informed and harmonized national legislation. Here, we clarify that “informed adaptive consensus” is not to be confused with “broad societal consensus”, which refers to all of society, including lay people. We have critiqued the use of this latter concept in Xafis and colleagues [6], as it erroneously conveys the notion that all of society can be included when, in fact, much of the world is necessarily excluded due to inequitable life circumstances.

### Would an international moratorium work?

The establishment of a moratorium would require a number of features to be addressed explicitly. These include the exact substance of the moratorium, i.e., what exactly is to be prohibited and what may be permitted, particularly in exceptional circumstances. It would also require the exact juridical, ethical, and legal bases and objectives for the moratorium as well as the term of the moratorium. In relation to the latter, by definition, a moratorium is a temporary suspension of an otherwise permitted activity. Moratoriums are therefore articulated for either fixed terms, usually with a clause allowing for extension by agreement, or predicated upon an event/circumstance that would trigger its determination or dissolution. In defining the trigger or determination event (if the moratorium were not to be for a renewable fixed term), justifications would have to be advanced for putting forward the proposed trigger or determination event and how this might serve the bases and objectives of the moratorium. As a result of this key feature of moratoriums, any notion of a permanent or open-ended moratorium cannot hold.

The final feature of moratoriums relates to the likely efficacy and impact of the proposed measure. Given the sovereign state principle in international law (i.e., that states have exclusive sovereignty over their territory), a moratorium would need to address issues regarding its ability to have real effect because, if it did not, it would simply result in another ineffectual political expression of intent. Additional considerations regarding its effect relate to a number of critically important considerations. The foremost among these is the “bottom-of-the-barrel” scenario—countries signing up to such a moratorium may find researchers moving off to more receptive climes with more “liberal” regulatory regimes with less oversight and restrictions. Would overly broad regulation or direction have a chilling effect on useful research in allied areas through discouraging researchers, for example, as a result of high administrative burdens, too many added complicated procedures, and having to put forward levels of justifications not required in other areas of research? Finally, most researchers would want to avoid the taint of unfounded “association by guilt” by a public or press that does not understand the vital difference between their work and that sought to be regulated.

### What about regulation through municipal legislation?

One approach is to rely on individual states to regulate at the national level through municipal legislation. However, this option does not necessarily promote the cause of international harmonization: In an ideal world, municipal law enacted by countries would be harmonized in the sense that they are at least roughly consistent with each other and have similarly common objectives. A detailed survey of the regulatory mechanisms in 18 countries found that while there is generally a ban on clinical applications of HGGE, some of these bans are not absolute, while other rules are “vague and fail to provide clear guidance as to what is allowed” ([7], p. 139). The lack of international harmonization is problematic; given the experience of human embryonic stem cell research, research resources may tend to eventually concentrate in jurisdictions that take a more relaxed or supportive approach to such research. Hence, safety and ethical concerns relating to genome editing are not adequately addressed with this option.

The greatest drawback of relying on municipal law at this stage is that there is no general clear consensus on precisely what should or should not be permitted in the name of research and in clinical application currently or in the future. The reality is that it is such early days yet in HGGE technology that we cannot know, or be agreed upon, from the ethical, social, as well as the scientific perspective, what kind of restrictive measures we want carved in legislative stone at this stage of development. Ideally, municipal law should reflect an established and mature body of ethical and scientific consensus, but the argument may be that we are currently just opening the debate and far from reaching any mature body of consensus. The act of legislation in most countries is ultimately at heart a political act—ethics and science may inform or be used to drive the legislative debate, but neither have an assured or primary place at the table. Legislators are first and foremost politicians: Science (and sometimes ethics) are often not the controlling premises for their actions. In its most innocent and defensible aspect, this political ranking of priorities may instead prioritize deeply held popular religious perspectives and economic and national interests in preserving the scientific advantages in a given field already established by the scientific or academic community in the country. Once enacted, the regulatory regime tends to ossify and be set in stone—repealing, or amending or “bringing up to date” a law that attracts strong political views on opposing sides of the legislative chamber may prove to be just as difficult as enacting it in the first place, particularly given the nature of HGGE, in which religious sentiments are likely to be a prominent theme in the public debate in some countries. Likewise, the disadvantages of overly precipitate and premature legislation based on an incomplete state of scientific knowledge are obvious.

### How about an international treaty or instrument instead?

Another approach may be to work toward the promulgation of international norms in an international treaty or instrument regulating the research and clinical use of HGGE. Again, as for muncipal law, the main obstacle to this normative approach is the current lack of consensus on the ethical, legal, and social dimensions of HGGE and how this may be best regulated and directed. A fundamental plank of international law is that all states are sovereign and equal, as enshrined in Article 2 of the United Nations (UN) Charter, which declares that “the Organization is based on the principle of the sovereign equality of all its Members.” This basically means that, with a very few exceptions (such as security matters within the purview of the Security Council when acting against another state, or crimes against humanity when acting against individual citizens of other states), no country is entitled to impress its law or will against another (in theory anyway). In short, countries are free to exercise their sovereign right to refuse to play in accordance with rules unpalatable to them.

Even when countries accede to a treaty, they are, in most cases, free to withdraw at any time. Even if, in fact, a country signs up to a treaty, it does not mean that the obligations and provisions of the treaty automatically become translated directly into municipal law enforceable by the municipal judicial authorities (unresolved international jurisprudential debates about whether monism (which postulates that once a country signs up to a treaty, the provisions of that external treaty automatically become part of the body of municipal law) or dualism (in which the provisions of the treaty have to be specifically translated into municipal law by the national legislature before it has any municipal effect) should be applied to international law in its relation to municipal law date back to the earliest roots of the concept of international law). It suffices to say, for our present purposes, that there is no jurisprudential agreement on this matter, and different countries take different (and, often, mixed and complexly nuanced) perspectives. And if a dualist country refuses to implement its international obligations under the treaty, in most cases, there is little that other countries can do—again, this is the sovereignty doctrine at work.

But as the United Nations General Assembly (UNGA) experience in the Declaration on Human Cloning amply demonstrates, the biggest obstacle in the way of a clear international treaty or instrument is the simple lack of agreement currently on what should be restricted (or what should be restricted in the way of research or in clinical applications), as well as the very different competing interests and first premises that different countries may bring to the debate. Many of those countries that refused to sign up to the Declaration on Human Cloning had communities of scientists already engaged in advanced research that would have been affected by the Declaration; for other countries, religious perspectives on human cloning and human embryonic stem cell research were the principal driving forces for their national positions on the debate. Similar divergences are likely to appear for HGGE.

Finally, the length of time required to negotiate and ratify an international agreement risks rendering the content of the agreement “obsolete” by the end of this process due to the rapid scientific developments. Governance mechanisms need to be adaptable and responsive, as highlighted in the recent WHO framework [3]. This requirement is a fundamental driver of an informed adaptive consensus, as it supports such responsiveness.

### Other practical options that might work?

It is often tempting to turn to law (whether municipal or international) to regulate spheres of human endeavor. But, instead of using the blunt and inflexible instrument of the formal law, working toward the establishment of a body of international norms outside of formal treaties may be an approach that can be used to address the tension between the need for international harmonization and the difficulty in making legally binding international law. This is in line with the trend since the start of the 21st century where international cooperation has moved away from legally binding international laws to nonlegally binding instruments [8]. In recent decades, we have seen the establishment or development of a consensus of norms in new and rapidly developing areas of biomedical technology and biomedical research that do not rely on harnessing the mechanisms of formal international law. Examples of such an approach include the following:

- recommendations issued by the International Committee of Medical Journal Editors (ICJME), which require adherence to standardized ethics guidance to publish in top ICJME journals. Hundreds of non-ICJME journals have also voluntarily adopted these guidelines [9];
- statements issued by the Human Genome Organization (HUGO), which have impacted on policies developed in the European Union [10] and on contributions such as the *Statement on Benefit Sharing* [11], which has been endorsed by WHO [12];
- the International Council for Harmonisation of Technical Requirements for Pharmaceuticals for Human Use (ICH) consortium initiative, which forms the basis of a harmonized worldwide framework for the conduct of clinical trials and has resulted in consensus guidelines, such as the ICH E6 Good Clinical Practice (GCP) [13]. Most technologically/economically advanced countries have incorporated one version or another of the ICH GCP E6 into their domestic regulatory requirements;
- the GA4GH Framework for Responsible Sharing of Genomic and Health-Related Data [14]. The framework was developed under the auspices of the GA4GH with collaborative input from a wide range of individuals and bodies around the world. A large number of national and international real-world genomics research programs implement the GA4GH frameworks and standards [15].

A key limitation of regulatory initiatives in both the international and municipal legal spheres is that only a very limited number of key stakeholders have a direct hand in the shaping of regulations. In the case of municipal law, legislators not only have a primarily political focus, but are usually also far removed from the actual experience and practice of the science involved, so that both the ethical as well as the scientific implications of developments must be received secondhand from the primary experts. At best, there is a risk of distortion or imperfect translation in such transmission; at worst, there is the possibility of deliberately slanted perspectives by less than scientifically objective experts. The ongoing political struggles over whether lockdowns are justified in the current pandemic is a painful reminder of such a reality. Likewise, the general rule in interstate relations is that only state actors are entitled to a place at the table: All other parties (such as nongovernmental entities or UN agencies) are permitted a peek in only on sufferance or agreement by the state actors concerned. But, in the initiatives that we have identified above, the fundamental principle is maximum inclusivity of the stakeholders most relevant to the issue at stake: participation of the many who are directly involved, and who have the greatest expertise, instead of a few far removed from the actual practice and impact of the science.

To this end, these initiatives have been deliberately fashioned outside of the established mechanisms for the creation of international law, and they demonstrate that state actors are not the only stakeholders who may influence or contribute to shaping the rules. The creation and willing uptake of these and other comparable initiatives illustrate that the views of academia, industry, patient lobbies, public interest groups, non-governmental organizations (NGOs), and other stakeholders exert influence and contribute in substantial ways to developing outputs likely to be palatable to a broader audience because they tend to be a product of consensus. There is, however, always an inherent risk in attempting to reach too widely for a consensus, as the result may be settling for a less meaningful lower common denominator.

In the HGGE space, entities with gatekeeping powers capable of considerable influence include biotechnology companies and fertility clinics, which have the power to refrain from offering products or services that are ethically problematic or likely to be used in an ethically problematic way; universities, funding foundations, international consortia and organizations, and WHO itself are other such entities. In addition, the top scientific and medical journals wield enormous normative power and influence in the shaping of norms through requirements they implement, as shown above. Gatekeepers such as professional bodies, scientists, and clinicians who are directly involved in the field are especially well positioned to understand which approaches are likely to result in the best outcomes. We believe that we should do more to explore working through nongovernmental gatekeepers such as those identified here. This approach also appears to be partially explored and supported in the recent NASEM report on HGGE [16] and is also supported in the newly released WHO framework [3].

## Conclusions

In sum, we propose that nonlegally binding instruments are the most promising approach to international governance of HGGE. One of the most important advantages of adopting such an approach that stresses the participation and product of the many, instead of the few, is that the product is one that is constantly being refined and articulated to address and reflect scientific developments as and when they appear: It is a gradual and flexible process in which international (as opposed to merely municipal) consensus can be gradually built up. In coming to an international consensus with their peers abroad, communities of scientists within a given country are also thereby afforded greater credibility than if they were lobbying alone with their national politicians. Politicians are also thereby quickly made aware of likely policy directions in competing jurisdictions. Non-state actors and participants directly involved in the science can move much more quickly and more expertly to formulate draft guidelines than legislators and state actors relying on secondhand information and bound by the rules of municipal and diplomatic protocols. In this way, scientists and ethicists alike can seize the initiative and provide guidance for the eventual formulation of municipal regulation, rather than the other way around. International and municipal laws have a place when the core consensus has been achieved and when the body of international expert opinion on the way forward is reasonably mature. Until then, all involved in science and ethics should consider what informal levers of power may be available to them and how these may be used to lead responsibly and ethically. A decision to halt any and all involvement in HGGE via a moratorium is not prudent, as it would delay the hard work we will ultimately have to engage in to find prudent solutions, and, importantly, it would bring to a halt an area of science that may have much to offer future generations.
